# The Natural Syntax of Local Coreference

**DOI:** 10.3389/fpsyg.2021.660296

**Published:** 2021-05-19

**Authors:** William O'Grady

**Affiliations:** Department of Linguistics, University of Hawai‘i at Mānoa, Honolulu, HI, United States

**Keywords:** coreference, anaphora, natural syntax, direct mapping, algorithm, processing, emergentism

## Abstract

Emergentist approaches to language are burdened with two responsibilities in contemporary cognitive science. On the one hand, they must offer a different and better understanding of the well-known phenomena that appear to support traditional formal approaches to language. On the other hand, they must extend the search for alternative explanations beyond the familiar languages of Europe and East Asia. I pursue this joint endeavor here by outlining an emergentist account for constraints on local anaphora in English and Balinese, with a view to showing that, despite numerous proposals to the contrary, the two languages manifest essentially the same system of coreference and that the system in question is shaped by processing pressures rather than grammatical principles.

## Introduction

The problem with emergence is that it is everywhere. Countless complex systems have properties that can be traced to the interaction of simpler factors, forces and events—everything from rush hour traffic to the evolution of species to the spread of viruses. As Elman et al. (1996, p. 2) note in *Rethinking Innateness*, one of the founding documents of emergentism, ‘the problem with this view in the past has been that, lacking a formal and precise theory of how such interactions might occur, talk of “emergent form” was at best vague. At worst, it reduces to hopeless mysticism.'

Now, though, emergence has a different challenge: it is widely recognized and acknowledged, but questions remain as to how its explanatory potential can be exploited for the types of problems confronting modern linguistics. At one time, the claim that the structure and acquisition of language are the product of emergence distinguished a novel line of scholarship from the then-dominant view that the human language faculty includes inborn grammatical principles. But the territory has since changed in two quite fundamental ways.

First, by the turn of the millennium, doubts had begun to surface about the nature of Universal Grammar (UG). Indeed, the doubters included Noam Chomsky himself, who launched a new research program that came to be known as “Minimalism.” Part of that program involved questioning the existence of the very domain-specific principles that had once been the *sine qua non* of UG.

There is no longer a conceptual barrier to the hope that the UG might be reduced to a much simpler form, and that the basic properties of the computational systems of language might have a principled explanation instead of being stipulated in terms of a highly restrictive language-specific format for grammars (Chomsky, 2005, p. 8).

This line of thinking culminated in the Strong Minimalist Thesis.

The optimal situation would be that UG reduces to the simplest computational principles, which operate in accord with language-independent conditions of computational efficiency (Chomsky, 2017, p. 296).

A second influential development is that as more scholars came to see the importance of emergence to the study of language, a variety of new perspectives have arisen, creating a more diverse field of play than had previously existed. Signs of this diversity, already evident in the ground-breaking volume *The Emergence of Language*, edited by MacWhinney (1999a,b), had broadened exponentially by the time the *Handbook of Language Emergence* (MacWhinney and O'Grady, 2015) was published a decade and a half later. The diversity came into full bloom at the 2019 symposium honoring the impact of Brian MacWhinney on language research (https://sites.google.com/view/macwhinney-symposium/home), at which almost 40 scholars offered numerous perspectives on what an emergentist theory of language might look like. Recent interest in emergentism has created what MacWhinney (2015, p. 9) has characterized as “an embarrassment of riches,” leading to the question of how students and scholars are to make sense of a landscape in which “all of these approaches fall under the general category of Emergentism.”

The section entitled The Strict Emergentist Protocol addresses this issue by bringing to the fore three ideas that have played a major role in work on linguistic emergentism and that together make up a protocol for studying the relationship between form and meaning. The sections entitled The Syntax of Coreference and Beyond Principle A focus on how these ideas contribute to an understanding of the syntax of coreference, which has long been assumed to favor UG-based approaches to language. As I will attempt to show, this assumption is ill-founded; indeed, there is even reason to think that the emergentist approach provides a *better* account for certain facts, including the curious patterns of co-reference found in various Western Austronesian languages. The section Making Sense of the Syntax of Anaphora offers a possible rationale for the syntax of anaphora that emerges from these facts.

Two provisos are in order before proceeding. First, consistent with the theme of this volume, the ideas that I put forward are quite programmatic, focusing on the outlines of what a theory of emergentist syntax might look like—a project that is developed in much more detail in O'Grady (2021). Second, in light of the need to establish the viability of emergentist approaches in a field largely dominated by theories of formal syntax, it is necessary to identify phenomena for which the two theories make different predictions. As often happens in such cases, this requires attention to a quite small set of critical sentences, at the expense of a larger survey of facts that might otherwise be called for.

## The Strict Emergentist Protocol

As acknowledged above, there are many imaginable varieties of emergentism and many ways to exploit its leading ideas in confronting the explanatory challenges presented by the study of language, including the two most central issues of all:

- Why do languages have the particular properties that they do?
- How are those properties acquired by children?

As a first step toward addressing these questions, I propose three ideas, each of which runs counter to standard assumptions within formal linguistics but which have been under consideration for some time in various lines of emergentist thought. The first idea challenges the existence of conventional syntactic structure, the second rejects the need for a grammar, and the third proposes that the operations required to bring together form and meaning in natural language are shaped mainly by processing pressures. Let us consider each in turn.

### Direct Mapping

It is a matter of consensus, from Aristotle to Elman to Chomsky, that language provides a way to map meaning onto form (typically a string of sounds), and vice versa.

A sentence is a spoken sound with meaning (Aristotle, cited by Everson, 1994, p. 91).

Grammars are complex behavioral solutions to the problem of mapping structured meaning onto a linear string of sounds (Elman et al., 1996, p. 39).

… every approach to language presupposes, at least tacitly, that a language determines a specific sound-meaning correlation (Chomsky, 2015, p. 93).

The question that must now be answered is thus clear: what are the mechanisms that bring together sound and meaning?

The predominant view in formal linguistics is that the relationship between form and meaning is mediated by syntactic representations (informally dubbed “tree structures”) whose signature feature is a hierarchical binary-branching architecture.

***Mediated mapping***


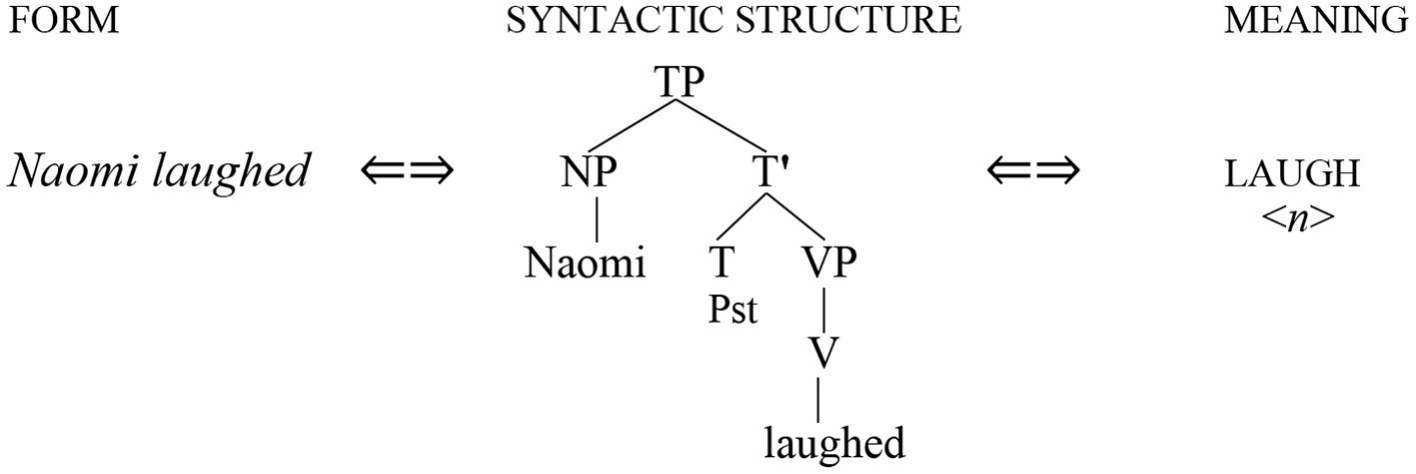


The thesis of mediated mapping is an essential assumption in the literature on generative grammar.

… the correlation of sound and meaning is mediated by syntactic structure … (Jackendoff, 2007, p. 3).

… any theory of [generative grammar] must assume the existence of a computational system that constructs hierarchically structured expressions … (Chomsky et al., 2019, p. 232).

In contrast, I propose to make the case for a direct relationship between form and meaning that does not require the mediation of syntactic structure.

***Direct Mapping***

The mapping between form and meaning does not require syntactic representations.

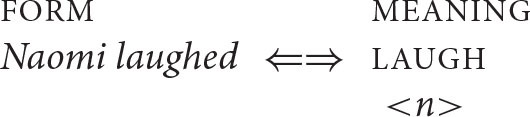


An idea along these lines has been explicitly championed in the emergentist literature for some time.

Only two levels of processing are specified: a functional level (where all the meanings and intentions to be expressed in an utterance are represented) and a formal level (where the surface forms appropriate for a given meaning/intention are represented). Mappings between the formal and functional levels are … direct (MacWhinney et al., 1984, p. 128).

[language] maps a string of words directly onto a semantic representation without the mediation of grammatical principles or syntactic structure (O'Grady, 2015, p. 102).

To avoid possible confusion, two clarifications are in order. First, the rejection of syntactic structure applies specifically to “tree structures.” It does not deny that speech involves words of particular types (nouns, verbs, etc.) that are inflected and linearized in particular ways. Second, I am not proposing that syntax can be dispensed with, only that it should be reconceptualized as the set of operations that map strings of words directly onto semantic representations and vice versa, as I will illustrate below.

### Algorithmic Orientation

A second issue with far-reaching consequences involves the particular level at which the mapping operations are investigated and described. Marr (1982) proposed three possibilities.

***Marr's three levels of analysis:***

- The computational level describes the goal(s) of the system, the information that it manipulates and the constraints that it must satisfy.
- The algorithmic/representational level describes the system in terms of the representations and data structures involved and the algorithms that manipulate these representations.
- The implementational/physical level addresses the question of how the system is physically realized (Marr, 1982; see also Johnson, 2017).

As Marr notes, generative grammar is a computational-level theory: it studies language as a system of knowledge, setting to the side the question of how that knowledge is put to work in the course of speech and comprehension.

a generative grammar … attempts to characterize in the most neutral possible terms the knowledge of the language that provides the basis for actual use of language by a speaker-hearer. When we speak of a grammar as generating a sentence with a certain structural description, we mean simply that the grammar assigns this structural description to the sentence… (Chomsky, 1965, p. 9)

On this view, the mechanisms that are required to produce and understand sentences—the subject matter of the theory of “performance”—are not part of the grammar *per se*, although they do interact with it.

[parsing and perception] have their own mechanisms, and can access unbounded external resources, but in doing so they surely access the generative mechanisms… (Chomsky, 2015, p. 95–96)

The Strict Emergentist Protocol shifts attention to the algorithmic level, where real-time processing occurs.

***Algorithmic Orientation***

Explanatory theories of language should focus on the algorithms that bring together form and meaning in the course of speech and comprehension.

In its strongest form, which I adopt here, an algorithmic orientation denies the existence of grammar in the sense of a cognitive system that assigns structural descriptions to sentences.

### Processing Determinism

A third issue now calls for attention: in the absence of syntactic structure and grammatical principles, we must ask what shapes the mechanisms that ensure the correct mapping between form and meaning—the ultimate focus of any explanatory theory of language. I propose that the key factor involves processing pressures.

***Processing Determinism***

The properties of algorithms are shaped by processing considerations.

Two types of forces seem to be in play—one internal and the other external.

Internal forces are focused on minimizing the cost of processing operations, which can be achieved in a variety of ways. Of most relevance to the topic of this chapter is the preference for form-meaning mappings that make the least demands on working memory, a strategy which has been explored in some detail in the previous emergentist literature and which will come into play here in the section entitled The Syntax of Coreference.

there is an advantage to reducing the burden on working memory, whatever its nature and whatever its capacity … the effects of this advantage can be discerned in the way that sentences are built (O'Grady, 1998, p. 6).

The conclusion I derive from much of the working memory literature, and from comparisons of different domain sizes within and across languages, is simply that the more items there are to process … the harder it is – i.e., … processing difficulty [in a given domain] increases where there are more forms and their properties to process and hold in working memory… (Hawkins, 2014, p. 232)

In contrast, external forces arise from factors manifested in experience, including the relative frequency of particular items and patterns in the speech of others. This too makes sense: the more frequently a word or pattern is heard and used, the stronger and more accessible the corresponding processing routine becomes.

Repeated exposure to a particular [linguistic] pattern … increases [the] speed and fluency of processing of the pattern (Bybee and McClelland, 2005, p. 396)

… the more frequently a construction is used, the easier it becomes to process (Imamura et al., 2016, p. 2).

Some emergentist work places a great deal of emphasis on the relevance of the frequency factor to phenomena ranging from language acquisition to typology (e.g., Ellis, 2006; Ambridge et al., 2015; Haspelmath, 2021). Although I acknowledge a role for input in shaping language and learning, I believe that its importance has been exaggerated in many cases and that internal processing pressures are the more powerful influence. I'll return to this matter at the end in the section entitled The Basics of an Emergentist Analysis.

### How Mapping Works

The three claims that make up the Strict Emergentist Protocol—direct mapping, algorithmic orientation and processing determinism—define a natural syntax for human language^1^.

***Natural Syntax***

The mapping between form and meaning is shaped and constrained by factors such as memory and processing cost that have a natural and well-established role in cognition, independent of language.

In order to illustrate how this conception of syntax might be implemented, it is first necessary to consider a notation for representing the forms and meanings upon which mapping algorithms operate. I will have little to say here about the representation of sound, for which a written string of words will stand as a proxy. Moreover, for the most part, I will make use of very simple semantic representations that contain little more than information about predicates and their arguments (predicates are represented in upper case and arguments in italicized lower case, along the lines illustrated above; see Kroeger, 2018, p. 67–68, among others, for a similar notation).

A full semantic representation must of course include a great deal of additional information (tense, aspect, modality, gender, definiteness, and the like). Nonetheless, the form-meaning mappings that underlie a very large number of syntactic phenomena, including coreference, appear to draw on little more than the spare representations illustrated in Table 1. For extensive discussion of this point, see O'Grady (2021).

**Table 1:** Representing sound and meaning.

**“Sound”**	**“Meaning”**	
*Naomi laughed*	LAUGH	
	<*n*>	(*n = Naomi*)
*Max teaches French*	TEACH	
	<*m f*>	*(m = Max; f = French*)

In order to lay the groundwork for what lies ahead, I will briefly outline three operations, or algorithms, which work together to map strings of words onto a corresponding semantic representation in an SVO language (↦ = “is mapped onto”).


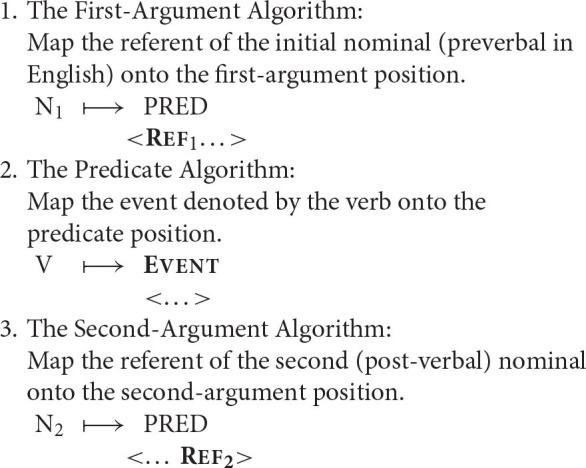


The example that follows illustrates how a sentence of English can be mapped onto a corresponding semantic representation.

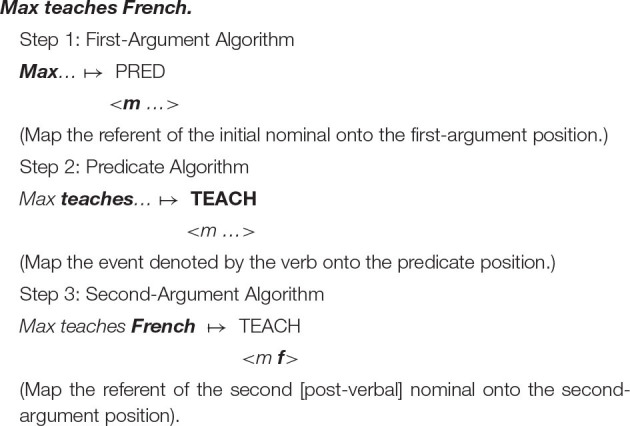

The order in which the algorithms are activated reflects and determines the arrangement of a sentence's component parts. As illustrated in Table 2, the same algorithms, applied in different orders, can yield different syntactic patterns.

**Table 2:** The order of activation of algorithms by word order type.

**SVO**	**VSO**	**SOV**
1. Map 1st argument	1. Map verb	1. Map 1st argument
2. Map verb	2. Map 1st argument	2. Map 2nd argument
3. Map 2nd argument	3. Map 2nd argument	3. Map verb

By making the algorithms sensitive to factors such as case marking, rather than (just) word order, it is possible to further extend the mapping options. These and other matters are discussed in detail by O'Grady (2021).

The study of coreference^2^ provides an opportunity to explore a different aspect of the mapping between form and meaning while at the same time probing a phenomenon of very significant import to our understanding of language.

… anaphora has not only become a central topic of research in linguistics, it has also attracted a growing amount of attention from philosophers, psychologists, cognitive scientists, and artificial intelligence workers… [It] represents one of the most complex phenomena of natural language, which, in itself, is the source of fascinating problems… (Huang, 2000, p. 1)

The next section sketches an outline of an emergentist account of this phenomenon, which will be extended in the sections Beyond Principle A and Making Sense of the Syntax of Anaphora.

## The Syntax of Coreference

The prototypical example of anaphora involves reflexive pronouns, whose interpretation is determined by an expression elsewhere in the sentence (the “antecedent”). Crucially, there are constraints on the positioning of the antecedent, as the following contrast illustrates in a preliminary way.

Marvin disguised himself.

^*^Himself disguised Marvin.

As a first and informal approximation, it appears that a subject can serve as the antecedent for a direct object, but not vice versa^3^.

When the subject and object are identical, we use for the latter a so-called reflexive pronoun, formed by means of self… (Jespersen, 1933, p. 111)

The contrast is made all the more interesting by the fact that this generalization appears to be universal.

[Basic] subjects in general can control reflexive pronouns [but not vice versa] (Keenan, 1976, p. 315).

… there appears to be no language in which the patient argument outranks the agent argument for the purposes of anaphora (Falk, 2006, p. 66).

Children receive remarkably little exposure to key patterns of anaphora. The data in Table 3 comes from a search that I did in the CHILDES corpus of speech to Adam, Eve and Sarah. The samples consist mostly of hour-long bi-weekly child-caregiver interactions over a period of many months: from 2;3 to 5;2 for Adam, from 1;6 to 2;3 for Eve, and from 2;3 to 5;1 for Sarah (of the 42 instances of reflexives that were uncovered, 19 simply expressed the meaning “alone,” as in *by himself* , *by itself* and so on).

**Table 3:** Number of reflexive pronouns in maternal speech.

	**Himself**	**Herself**	**Itself**	**Themselves**
Adam	14	1	4	0
Eve	16	0	2	0
Sarah	2	1	1	1
Total	32	2	7	1

Uncontestably, input has an important role to play in linguistic development, but its usefulness needs to be measured against each component of the three-part puzzle that language learners confront every time they encounter a new word: what is its form, what is its meaning, and what is its syntax?

A child's exposure to a handful of reflexive pronouns may well allow her to identify the form that reflexive pronouns take (*him* + *self* , *her* + *self* , etc.). It may even give her enough information to identify an important component of their meaning: *himself* refers to a male human, *herself* refers to a female human, and so on. However, the syntax of these items is another matter. Given that the absence or infrequency of a particular pattern does not suffice to ensure its unacceptability (e.g., Yang, 2016, p. 143), why should mere exposure to a sentence such as *He hurt himself* lead a child to automatically reject patterns such as the following?

^*^His sister hurt himself.

^*^He said she hurt himself.

As a large number of experimental studies have demonstrated, children are remarkably successful at avoiding this sort of overreach. Indeed, they typically use and interpret reflexive pronouns correctly from the earliest point at which they can be tested.

Children display adultlike comprehension of sentences including reflexives from about 3 years and produce such sentences spontaneously from about 2 years. Children … can compute the local domain and, within this, determine the antecedent (Guasti, 2002, p. 290).

The challenge for an emergentist approach to the syntax of coreference is thus two-fold. On the one hand, it must offer an account for coreference asymmetries that does not require grammatical principles or syntactic structure. On the other hand, it must also provide an explanation for how children are able to master the relevant contrasts so quickly, based on so little exposure to their occurrence in adult speech. I will begin by proposing an algorithm for interpreting reflexive pronouns, locating it in the larger theoretical landscape and illustrating its functioning in a representative range of cases.

### The Basics of an Emergentist Analysis

Two generalizations define the interpretation of reflexive pronouns in English and many other languages.

- The reflexive pronoun requires a “local” antecedent—roughly speaking, an antecedent in the same clause.
- The antecedent must be in some sense more “prominent” than the reflexive pronoun, consistent with the observation above that a subject (agent) can serve as antecedent for a direct object (patient), but not vice versa.

In the literature from the last 50 years or so, there have been just two basic ideas about how to characterize the prominence asymmetry.

One approach, embodied in Principle A of Universal Grammar, exploits the architecture of syntactic structure. Its key claim is that reflexive pronouns look to a higher (“c-commanding”) antecedent for their interpretation^4^.

***Principle A*** (paraphrased)

A reflexive pronoun must have a c-commanding antecedent in the same clause (based on the binding theory proposed by Chomsky, 1981, p. 188).

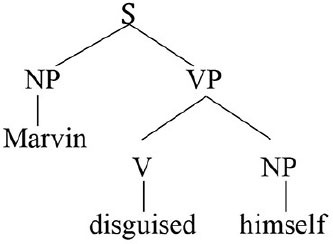


The c-command relation permits a structural definition of prominence: an expression can serve as antecedent for the reflexive pronoun only if it occupies a higher position in syntactic structure, as in the example directly above.

The second approach makes use of argument structure to capture the asymmetries underlying coreference. This can be done in a variety of ways. One popular idea is to arrange arguments in a hierarchy of grammatical relations, with the least oblique relation in the top (leftmost) position.

Subject < Primary object < Secondary object < Other complements

An anaphor must have a less oblique co-argument as its antecedent, if there is one (Pollard and Sag, 1992, p. 266).

Another idea makes use of a hierarchy of thematic roles, for which the literature offers various possibilities (for a review, see Rappaport Hovav and Levin, 2007). One early proposal looks like this.

Agent < Location, Source, Goal < Theme

A reflexive pronoun cannot thematically outrank its antecedent (Jackendoff, 1972, p. 148; see also Pollard and Sag, 1992, p. 297–99).

Both the relational hierarchy and the thematic-role hierarchy correctly license patterns of coreference in which the subject/agent serves an antecedent for a reflexive pronoun that function as a direct object/patient. Moreover, as desired, both also rule out patterns in which the reverse relationship holds (e.g., ^*^Himself disguised Marvin).

The approach that I propose is based on argument structure, although without reference to either grammatical relations or thematic roles *per se*. Instead, I focus entirely on the manner in which the arguments are ordered and organized relative to each other within argument structure.

A key initial assumption is that the agent is universally and invariably the first argument of a transitive verb^5^ (ag = agent; pat = patient).


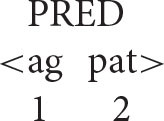


This makes sense from the processing perspective that underlies natural syntax. As the instigator of the action denoted by the verb, the agent is “the head of the causal chain that affects the patient” (Kemmerer, 2012, p. 50; see also Talmy, 1988, p. 61; Croft, 1991). Consistent with this observation, patienthood typically entails prior agency: an entity cannot become a patient until an agent has acted upon it (Bornkessel-Schlesewsky and Schlesewsky, 2009, p. 41). For instance, in the event described by the sentence *The students painted the house*, the patienthood of the house depends on prior action by the painters. At the level of event conceptualization, then, the agent is clearly ontologically prior and in this sense counts as the first argument, consistent with its traditional position in argument structure.

With this understanding of the organization of argument structure in place, it is now possible to formulate the following algorithm (α = the antecedent, *x* = the anaphor).

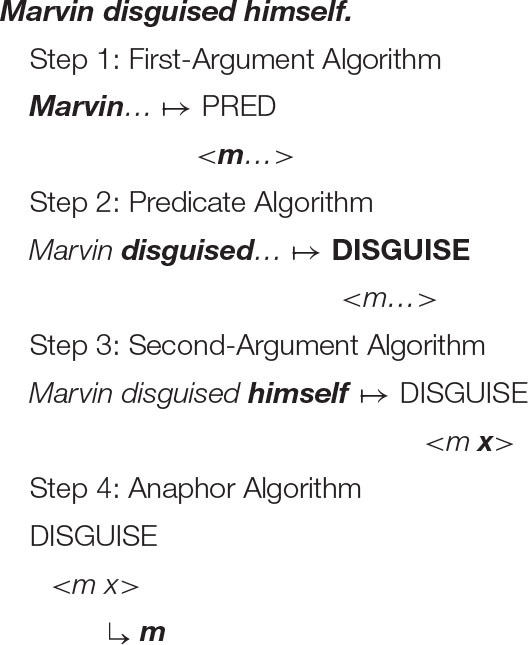

***Anaphor Algorithm***


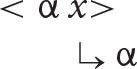


The interpretive operation embodied in this algorithm has three key properties:

- It applies to the semantic representation built by the mapping operations exemplified in the section How Mapping Works.
- It is triggered by the presence of a referential dependency (represented here as *x*) that is introduced by a reflexive pronoun.
- It resolves the referential dependency by associating it with a prior co-argument, represented in the algorithm by the symbol α.

A concrete example appears in the box above.

The first three steps map the words in the sentence directly onto a corresponding semantic representation, without the mediation of syntactic structure or grammatical rules. In the fourth and final step, the just-encountered reflexive pronoun receives an interpretation thanks to the Anaphor Algorithm, which links it to the verbal predicate's first argument, *Marvin*.

The Anaphor Algorithm has a quite obvious natural motivation: its job is to resolve referential dependencies immediately and locally, in response to internal processing pressures. At the point where the reflexive pronoun is encountered and identified as the verb's second argument, only the verb's first argument is immediately available to resolve the referential dependency.

Marvin disguised himself.


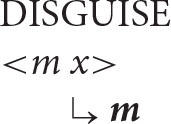


There is therefore just one option for interpreting the reflexive pronoun—the desired result.

Herein lies an attractive explanation for the ease with which children acquire the syntax of anaphora. Indeed, in a way, there is nothing for children to acquire; they have only to surrender to the natural impulse to minimize processing cost. The consequence of that impulse is the immediate resolution of the referential dependency by selecting the nearest possible antecedent—a prior co-argument. In other words, all children need to do is as little as possible.

No one should be unhappy about this, not even the proponents of usage-based development who place their bets entirely on the availability of generous amounts of friendly input.

Despite the daunting scope of linguistic phenomena begging an explanation, usage-based theories of language representation have a simple overarching approach. Whether the focus is on language processing, acquisition, or change, knowledge of a language is based in knowledge of actual usage and generalizations made over usage events (Ibbotson, 2013, p. 1; see also Tomasello, 2009; Lieven, 2014, among many others).

The enormity of this challenge should not be underestimated. Attention must be paid to frequency effects involving not only tokens but also yet-to-be-defined types, at levels of analysis ranging from the very concrete to the highly abstract (Ambridge et al., 2015). Overgeneralizations have to be identified and corrected (e.g., Boyd and Goldberg, 2011). Distributional tendencies require careful assessment to determine whether they are robust enough to support a useful generalization and, if so, how many exceptions can be tolerated before a revision is required (Yang, 2016). And so on.

All in all then, we should be more than pleased if significant pieces of language emerge for free in response to processing pressures. Indeed, in a theory of natural syntax, input-dependent usage-based learning is no more desirable than Universal Grammar. One is too difficult, and the other is too easy. Emergence is just right—a modest amount of input interacting with natural cost-driven preferences and restrictions.

### Some Basic Contrasts

On the assumption that the presence of a reflexive pronoun automatically triggers the Anaphor Algorithm, there is a natural account for the unacceptability of sentences like the one below.

^*^Himself disguised Marvin.

(compare: Marvin disguised himself).

Here, the usual operations produce a semantic representation in which the reflexive pronoun is the first argument, for which (by definition) there can be no prior co-argument.


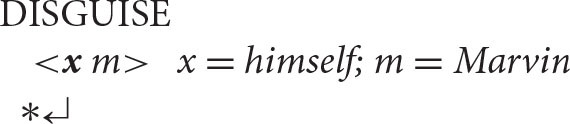


As a result, the Anaphor Algorithm is unable to do its work and the referential dependency is left unresolved, disrupting the mapping between form and meaning.

The next sentence illustrates another classic contrast—the antecedent for the reflexive pronoun in the following sentence has to be Marvin's brother, not Marvin.

[Marvin's brother] disguised himself.

This fact follows straightforwardly from the Anaphor Algorithm. As illustrated below, the prior argument is *Marvin's brother*, which is therefore automatically selected as the antecedent for *himself* .


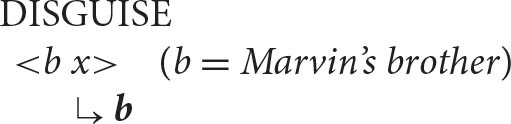


A third key contrast involves biclausal patterns such as the one below, in which there appear to be two potential antecedents for the reflexive pronoun.

Harry thinks [Marvin disguised himself].

On the processing account, the only permissible interpretation is the one in which the referential dependency introduced by the reflexive pronoun is resolved by the referent of *Marvin*, its co-argument. This is exactly the result guaranteed by the Anaphor Algorithm.


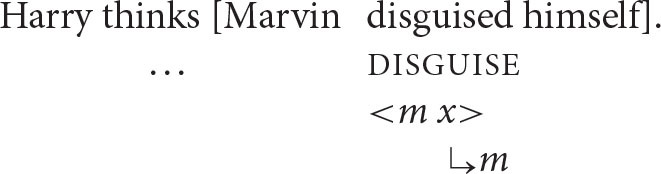


### Priority vs. Linear Order

The organization of argument structure is often reflected, as least loosely, in a language's canonical word order: agents are pronounced before patients in transitive clauses in more than 95% the world's languages (e.g., Dryer, 2011). However, a very small percentage of languages manifest the reverse order, apparently reflecting application of the Second-Argument Algorithm before the First-Argument Algorithm^6^. Malagasy, a language with verb–object–subject order, is a case in point.


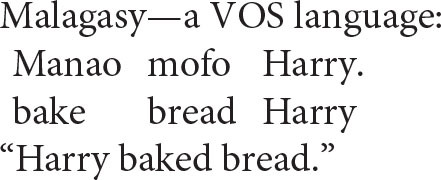


This does not matter for the syntax of coreference, however, since the Anaphor Algorithm operates on the semantic representation, not on the corresponding string of words. A striking illustration of this point comes from the following Malagasy sentence, in which the reflexive pronoun precedes its antecedent (the examples that follow are from Keenan, 1976, p. 314–315).


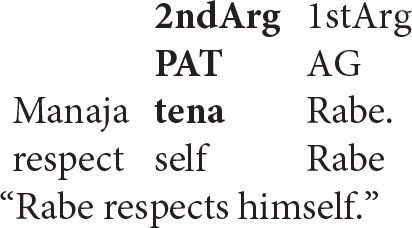


Word order notwithstanding, the patient (here a reflexive pronoun) occupies its usual second position in argument structure. Its interpretation can therefore be determined by reference to the agent, which occupies a prior position in argument structure, despite its position in the spoken form of a VOS sentence.


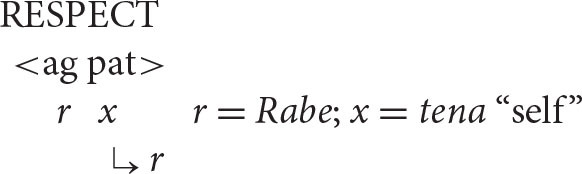


As predicted, the reverse pattern is unacceptable.


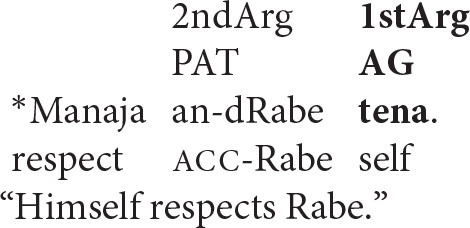


Here, the intended antecedent (the patient argument, *Rabe*) precedes the reflexive pronoun in the string of words that make up the sentence. But this is irrelevant: because the agentive reflexive pronoun is associated with the *first-argument position*, there is no prior argument to which it can look for its interpretation.


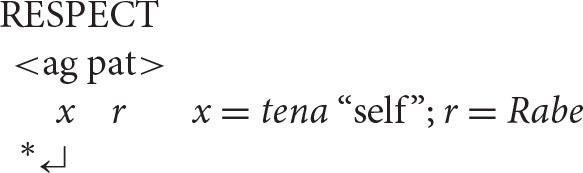


Examples like these confirm two key points.

- the computation of coreference takes place in argument structure, not in the string of words produced by the speaker and heard by the listener.
- the organization of argument structure can be independent of the order in which a sentence's words are arranged in its spoken form.

## Beyond Principle A

In all the cases considered to this point, the Anaphor Algorithm yields results comparable to those offered by Principle A. This is itself quite striking; it is more than a little surprising that a principle long used to illustrate the need for Universal Grammar and intended to apply to abstract syntactic structures could be challenged by an algorithm shaped by processing pressures and designed to help map a string of unstructured words directly onto a meaning.

It is tempting to wonder whether there might be cases of coreference for which *only* the Anaphor Algorithm offers an empirically successful account. A curious and little studied pattern of coreference found in a group of Austronesian languages offers a unique opportunity to explore this possibility. The key observation that has been made for these languages is that their system of anaphora defines prominence in terms of thematic roles. One language of this type, on which I will focus here, is Balinese, which is spoken by 3.3 million people on the island of Bali in Indonesia (similar systems are found throughout the Philippines; see, for example, Bell, 1976, p. 30 and 157; Schachter, 1976, p. 503–504; Andrews, 1985, p. 62–63; Kroeger, 1993).

### Reflexive Pronouns in Balinese

Balinese exhibits an intriguing syntax built around a system of symmetrical voice. The signature feature of this system, whose presence has been detected in dozens of Western Austronesian languages, is the co-existence of two competing transitive patterns, one highlighting the agent and the other elevating the prominence of the patient (Himmelman, 2002, p. 14; Chen and McDonnell, 2019, p. 14)^7^


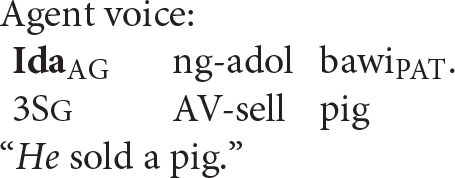



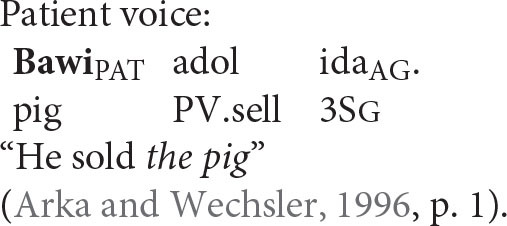


Wechsler and Arka (1998) show that the preverbal NP in both voice patterns is the “subject” in that only it can undergo operations such as relativization, raising, control, and extraposition—all common tests for subjecthood. Based on standard assumptions about syntactic structure in generative grammar, the agent should therefore occupy the structurally highest position in an agent voice pattern and the patient should occur in that position in a patient voice construction.


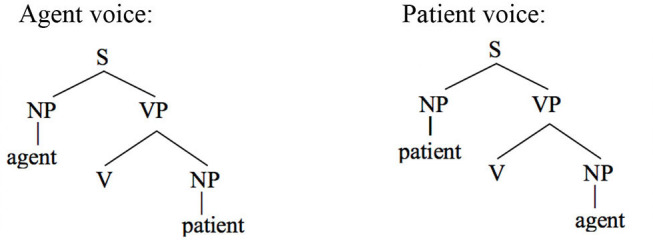


This leads to the following prediction about coreference in a theory that includes Principle A and therefore requires that a reflexive pronoun have a structurally higher antecedent.

***Predictions of Principle A***

- The agent argument should be able to serve as the antecedent of a patient reflexive in the agent voice.
- The patient argument should be able to serve as the antecedent of an agent reflexive in the patient voice.

In contrast, the Anaphor Algorithm creates a very different set of expectations. The starting point for this line of reasoning is the premise that the agent voice and patient voice patterns are both transitive and therefore have the same argument structure, with the agent as first argument and the patient as second argument.


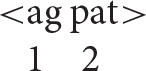


This leads to the following two predictions.

***Predictions of the Anaphor Algorithm***

- The agent argument should be able to serve as antecedent for a patient reflexive in both voice patterns.
- The patient argument should not be able to serve as antecedent for an agent reflexive in either voice pattern.

Let us consider the success of each prediction.

### Testing the Predictions

#### Co-reference in the Agent Voice

Coreference in the Balinese agent-voice pattern closely resembles what we see in its English counterpart (the Balinese data in this section is drawn from the pioneering work of Arka and Wechsler, 1996; Wechsler and Arka, 1998. Both *ida* and *ragan idane* are gender-neutral, but I will translate them as *he* and *himself* for the sake of simplicity).


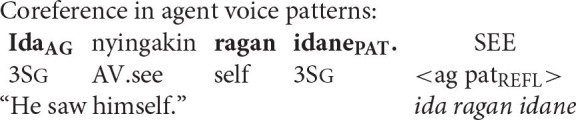


The acceptability of coreference in this pattern complies with the prediction of Principle A, since the agent argument (the antecedent) is higher in syntactic structure than the reflexive pronoun.


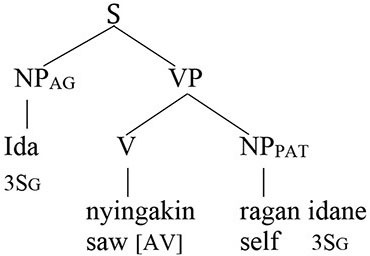


And, of course, it also complies with the prediction of the Anaphor Algorithm, since a patient reflexive can look to a prior agent argument for its interpretation.


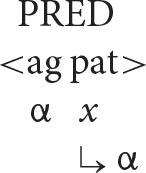


In other words, the two analyses make the same predictions about coreference in agent voice patterns, and both are correct. However, matters are very different when we consider coreference in patient voice patterns.

#### Co-reference in the Patient Voice

The pattern of coreference illustrated in the patient voice construction below offers a decisive insight into the true syntax of anaphora.


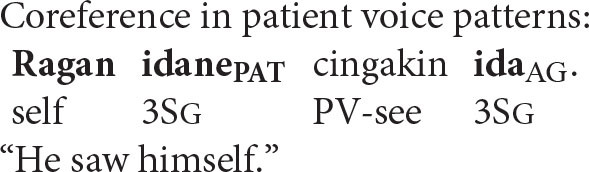


Given that Balinese is an SVO language (see the section Reflexive Pronouns in Balinese), the reflexive pronoun in the above sentence occurs in a higher structural position than its antecedent. Principle A therefore predicts that the sentence should be unacceptable.


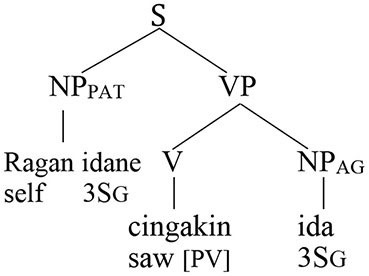


In contrast, the Anaphor Algorithm predicts that the sentence should be well-formed, since—regardless of word order—the patient is located in the second-argument position and is therefore able to look to the prior agent argument for its interpretation.


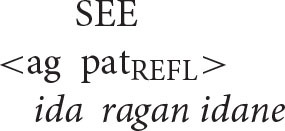


Crucially, the sentence is acceptable on the intended interpretation^8^.

The Anaphor Algorithm makes a further prediction: the patient-voice pattern below should be *unacceptable*.


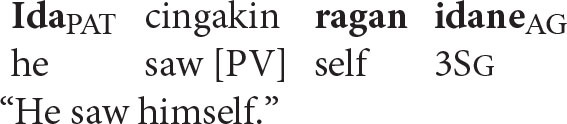


The antecedent (the patient argument *ida*) precedes the reflexive pronoun in this pattern and is higher in syntactic structure, perfectly positioned for the type of referential dependency required by Principle A.


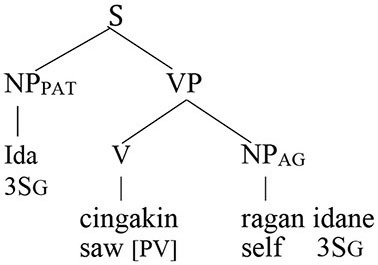


But this shouldn't matter if the Anaphor Algorithm is correct. Because *ragan idane*, the agent, occupies the first position in argument structure, there is no prior argument to which it can look for its interpretation.


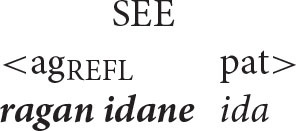


The sentence should therefore be uninterpretable and hence ill formed. This prediction is correct; the sentence is indeed unacceptable.

In sum, the facts from Balinese suggest that coreference in that language is not sensitive to syntactic structure. Rather, its unusual patterns of anaphora reflect the same algorithm that regulates coreference in English—an interpretive procedure that is shaped by the need to minimize processing cost. Moreover, consistent with proposals made by a long series of scholars, including Jackendoff (1972), Pollard and Sag (1992), and Wechsler (1998), coreference asymmetries are best characterized in terms of argument structure rather than syntactic structure. This is the very type of outcome predicted by the Strict Emergentist Protocol outlined in the section of that name.

## Making Sense of the Syntax of Anaphora

If the ideas I have been outlining are on the right track, the syntax of coreference appears to be organized around a simple intuition: an anaphor must look to a prior co-argument for its interpretation. Consistent with the idea that referential dependencies are computed and resolved in the semantic representation, priority is defined in terms of the organization of argument structure, not word order. Thus, English, Malagasy, and Balinese all have strikingly similar systems of anaphora despite differences in the ordering of pronouns relative to their antecedents in the spoken form of particular sentences.

One way to make sense of the system of anaphora that I have proposed is to consider the possibility that sentence planning is aligned with the perceived structure of the event that is to be expressed. In the case of a transitive action, the cognitive path begins with an agent and proceeds from there to the next argument, creating the conditions for a patient reflexive to derive its reference from a prior agent argument.

**Table:** 

**Transitive pattern (*Marvin disguised himself*)**			
***Plan ⇒***	***Event***	***1st argument***	***2nd argument (Refl)***
	DISGUISE ⇒	DISGUISE ⇒	DISGUISE
	<…>	<*m*…>	<*m **x***>
		AG	AG PAT
			↳*m*

This fits well with MacDonald's (2013) idea that the computational burden of planning and producing utterances promotes choices that reduce processing cost. In the case of anaphora, cost arises from the need to resolve a referential dependency, which can be facilitated by having the argument that introduces the referent in a position in argument structure prior to that of its pronominal co-argument.

The same reasoning can be applied to more complex argument structures, such as those associated with ditransitives.


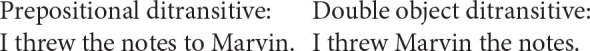


Two different argument structures seem to be in play here.

[There is] an operation that takes a verb with a semantic structure containing “X causes Y to go to Z” and converts it to a verb containing a structure “X causes Z to have Y” (Pinker, 1989, p. 82).

… the double object construction requires the semantics of caused possession and the *to*-dative construction requires the semantics of caused motion (Yang, 2016, p. 191).

In other words, in the prepositional pattern, the speaker acts on the notes by transferring them to Marvin. On this interpretation, the patient (*the notes*) is the second argument and the goal (*Marvin*) is the third argument, giving the representation depicted below (go = goal).


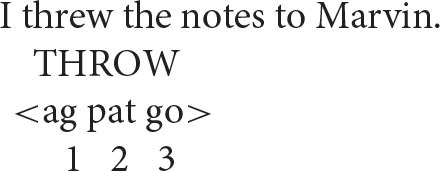


In the double object ditransitive, in contrast, the speaker acts on Marvin by having him receive the notes. Thus, in this pattern the goal is the second argument and the patient the third.


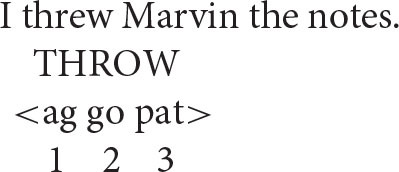


On this view, then, there is no fixed thematic-role hierarchy for patient and goal arguments. Rather, they can be ordered in different ways relative to each other, depending on how the event to which they contribute is conceptualized. However, the Anaphor Algorithm remains essentially the same, requiring that a reflexive pronoun have a prior antecedent in argument structure.


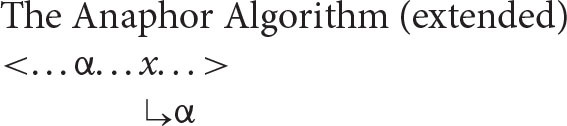


All of this leads to an important prediction about coreference: the patient argument should be able to serve as an antecedent for the goal argument in the prepositional pattern, and the opposite should be true in the double object pattern.


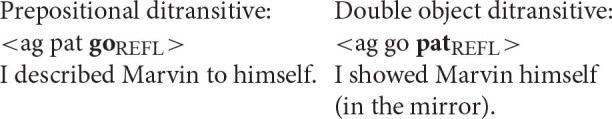


By the same reasoning, anaphoric dependencies that run in the opposite direction should not be acceptable.


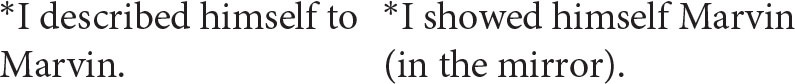


These facts suggest that the “flow” of argument structure proceeds one way in the case of prepositional ditransitives (ag–pat–goal) and another way in the case of double object ditransitives (ag–goal–pat).

**Table:** 

**Prepositional ditransitive (*I described Marvin to himself*):**				
**Plan ⇒**	***event***	***1st argument***	***2nd argument***	***3rd argument (Refl)***
	DESCRIBE ⇒	DESCRIBE ⇒	DESCRIBE ⇒	DESCRIBE
	<…>	<*i* …>	<*i m* …>	<*i m **x***>
		AG	AG PAT	AG PAT GO
				↳*m*

**Table:** 

**Double object ditransitive (*I showed Marvin himself in the mirror*):**				
**Plan ⇒**	***event***	***1st argument***	***2nd argument***	***3rd argument (Refl)***
	SHOW ⇒	SHOW ⇒	SHOW ⇒	SHOW
	<…>	<*i*…>	<*i m* …>	<*i m **x***>
		AG	AG GO	AG GO PAT
				↳*m*

Importantly, there is independent evidence that the two argument-structure patterns differ in the proposed way. The key insight comes from idioms, which typically consist of a verb and its “lowest” argument (O'Grady, 1998). Consistent with this observation, we find idioms such as the following.

Prepositional ditransitive—the goal is the third argument:

I **threw** Marvin **to the wolves**.

“I sacrificed Marvin to further my own interests.”

Double object ditransitive—the patient is the third argument:

I **threw** Marvin **some crumbs**.

“I made a minor concession to Marvin to placate him.”

As illustrated here, the idiom in the prepositional pattern consists of the verb and its goal argument (*to the wolves*), whereas the double object idiom is made up of the verb and its patient argument (*some crumbs*). This is exactly what one would expect if, as proposed, the third argument corresponds to the goal in the first pattern and to the patient in the second pattern.

In sum, we see in ditransitive patterns the same underlying forces that shape anaphoric dependencies in their simpler transitive counterparts. Put simply, coreference is managed in the course of sentence planning by reserving the use of reflexive pronouns for situations in which there is a prior co-argument from which they can derive their interpretation.

## Conclusion

The principles that generative grammar uses to regulate coreference are widely acclaimed for their descriptive success and have come to be a showcase example of Universal Grammar—its “crowning achievement” according to Truswell (2014, p. 215) and a “window onto the mind” according to others (Huang, 2000, p. 16). Anaphora does indeed provide a potential glimpse into the language faculty, but what it reveals is arguably not Universal Grammar.

On the view outlined in this chapter, neither grammatical principles nor syntactic structure enters into the computation of coreference. Instead, the interpretation of reflexive pronouns is shaped by processing pressures that promote the rapid resolution of referential dependencies—the very requirement embodied in the Anaphor Algorithm. Put simply, coreference has a natural syntax.

## Data Availability Statement

The raw data supporting the conclusions of this article will be made available by the authors, without undue reservation.

## Author Contributions

The author confirms being the sole contributor of this work and has approved it for publication.

## Conflict of Interest

The author declares that the research was conducted in the absence of any commercial or financial relationships that could be construed as a potential conflict of interest.

## Footnotes

^1^As used here, the term “natural” has the sense adopted in work on natural phonology (e.g., Donegan and Stampe, 1979) and natural morphology. As Dressler (1999, p. 135) suggests, the term “is synonymous to cognitively simple, easily accessible (especially to children), elementary and therefore universally preferred, i.e., derivable from human nature”.

^2^The terms “coreference,” “anaphora,” and “binding” overlap in their meaning and are often used interchangeably. The phenomena on which I focus in this chapter are mostly instances of coreference in that they take referring NPs rather than quantified expressions as their antecedents.

^3^In the spirit of Jespersen, I use the terms “subject” and “direct object” solely for the purposes of descriptive convenience; they have no technical standing in the emergentist theory I propose.

^4^Although first proposed four decades ago, this principle continues to have a wide currency in the literature and is a standard starting point for the study of coreference in textbooks (e.g., Carnie, 2013); for a more recent version of Principle A, see Reuland (2018). Haspelmath (forthcoming) offers a comparative overview of reflexive constructions in the world's languages.

^5^Passivization has the effect of downgrading the agent argument.

^6^This option shows up in many languages, including the very wide range of SOV languages that permit OSV patterns as an alternative word order (see O'Grady, 2021, p. 51ff).

^7^Patient voice patterns should not be confused with passives. In contrast to what one would expect in a passive, the agent argument is generally required in the patient voice, where it appears without the oblique marking typically found on agents in passive patterns the world over (e.g., a preposition equivalent to *by*). Even more striking is the fact that Balinese *has* a passive pattern, with morphosyntactic properties very different from those of the patient voice—including a passive prefix (*ka-*) and an optional agent that is introduced by an oblique marker (*antuk*).

^8^As Wechsler and Arka (1998, p. 407) note, this pattern requires that the antecedent be a pronoun rather than a lexical NP. Levin (2014) proposes an analysis in the generative framework to accommodate the Balinese facts, but only by substantially modifying the nature of anaphora and increasing the abstractness of the syntactic representations required for his hypothesis.
